# Supplementary material for: Competing Endogenous RNA Regulatory Networks of hsa_circ_0126672 in Pathophysiology of Coronary Heart Disease
Source: Genes (Basel). 2023 Feb 22;14(3):550. doi: 10.3390/genes14030550 (PMC10047999; doi:10.3390/genes14030550)
Supplement: Supplementary file 1 [file genes-14-00550-s001.zip › Figure S1-S3.pdf]

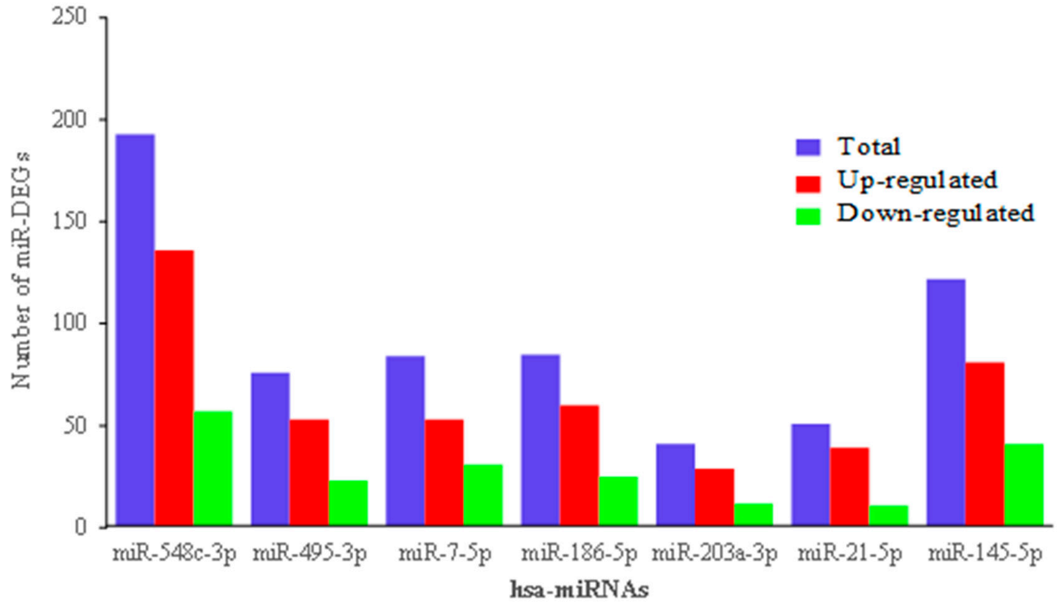

Figure S1: Number of CHD related differentially expressed genes potentially regulated by individual miRNAs.

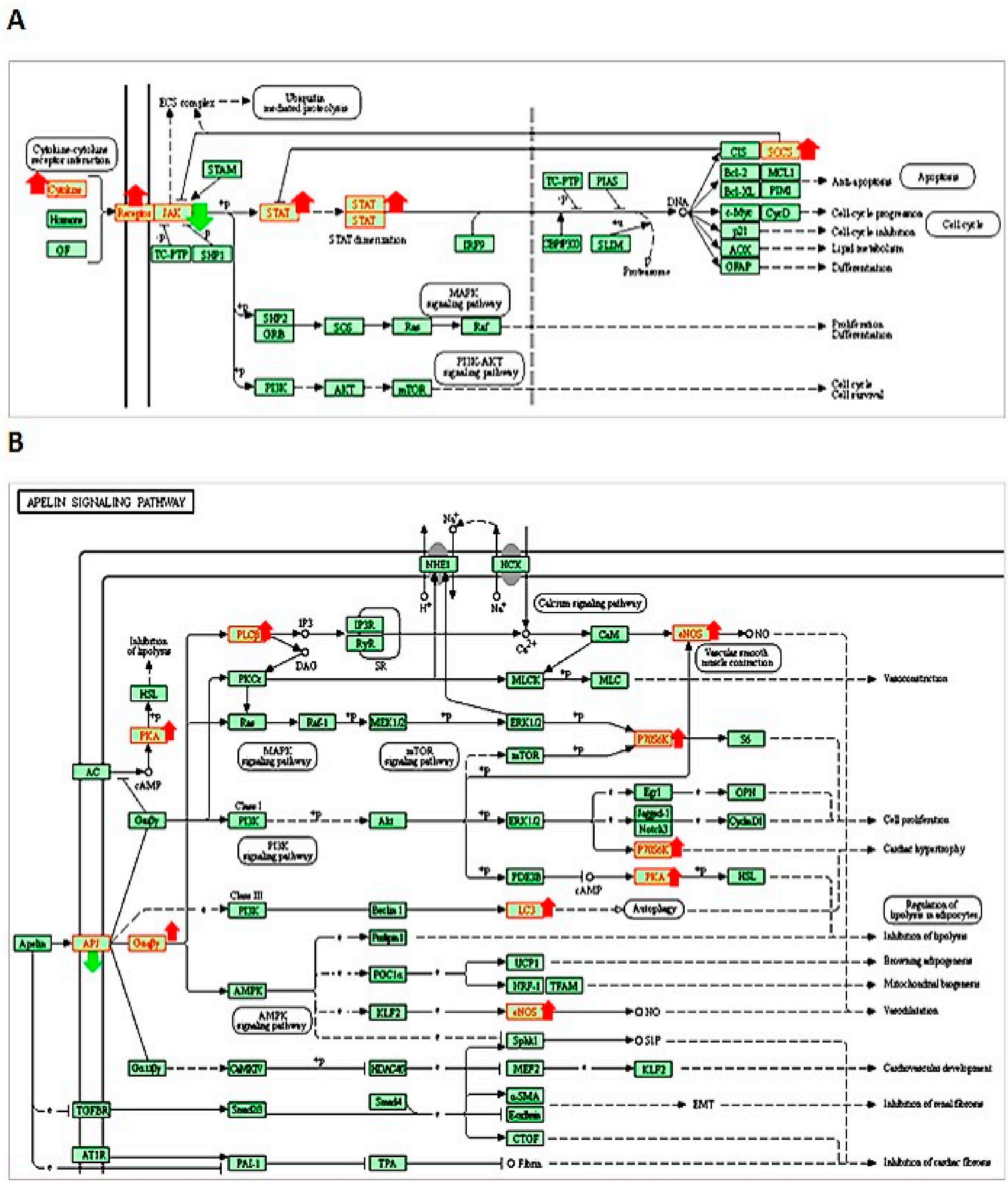

Figure S2. Involvement of miR-DEGs in the regulation of signaling pathways. A: JAK/STAT signaling pathway, B: Apelin signaling pathway. Red arrow indicates up-regulated gene, while the green arrow indicates down-regulated genes.

miRNA & length	Gene & length	Binding position	MFE (kcal/mol)	miRNA:mRNA Duplex Binding Pattern
miR-186-5p 22	GNB4 6315	1722	-27.8	target 5' G A UCU A U 3'<br>GGC CC GAGGGGAGUUC UUG<br>UCG GG UUCCUCUUAAG AAC<br>miRNA 3' UU A 5'
miR-186-5p 22	IL6ST 9027	8294	-27.0	target 5' U CAGUGA UU G 3'<br>AGCCCAAAGG GAGAGU UCUU<br>UCGGGUUUUC CUCUUA AGAA<br>miRNA 3' AC 5'
miR-145-5p 23	GNAQ 6882	468	-28.7	target 5' C CUCGGCGGCCACCGC G C GA G 3'<br>GGGGG U CCU GGGGGAGC GGGC<br>UCCCU A GGA CCCUUUUG CCUG<br>miRNA 3' A A 5'
miR-145-5p 23	SOCS2 2606	339	-27.9	target 5' C CUCG UUCGCACU UCAAGGAA G 3'<br>AGGGA UUUUGGGA GACU GGAC<br>UCCCU AGGACCCU UUGA CCUG<br>miRNA 3' A U 5'
miR-145-5p 23	RPS6KB1 5428.00	1071	-38.8	target 5' U GCUGG C G 3'<br>GGGA UCCUGGGGA GCUGGA<br>CCCU AGGACCCUU UGACCU<br>miRNA 3' U A U G 5'
miR-145-5p 23	NOS1 12007.00	618	-31.4	target 5' U U AG G U A 3'<br>GGGAUUUCUGG GA GAGC GG C<br>CCCUAAGGACC CU UUUG CC G<br>miRNA 3' U A U 5'
miR-145-5p 23	GABARAPL1 965.00	370	-27.2	target 5' C UG AU A 3'<br>AGGG CCUG CUGGAC<br>UCCC GGAC GACCUG<br>miRNA 3' UAA CCUUUU 5'
miR-145-5p 23	APLNR 3660	3073	-34.9	target 5' U UAGG G 3'<br>GGGGA UCUGGGAA GACUGGA<br>UCCCU GGACCCUU UUGACCU<br>miRNA 3' AA G 5'

Figure S3: mRNA-miRNA hybrids characteristics showing the minimum free energy (MFE) of the duplexes and binding pattern at predicted target sites.
